# Anthrax of the Gastrointestinal Tract

**DOI:** 10.3201/eid0807.020062

**Published:** 2002-07

**Authors:** Thira Sirisanthana, Arthur E. Brown

**Affiliations:** * Chiang Mai University, Chiang Mai, Thailand; † Armed Forces Research Institute of Medical Sciences, Bangkok, Thailand

**Keywords:** anthrax, anthrax classification, anthrax epidemiology, anthrax diagnosis, bioterrorism

## Abstract

When swallowed, anthrax spores may cause lesions from the oral cavity to the cecum. Gastrointestinal anthrax is greatly underreported in rural disease-endemic areas of the world. The apparent paucity of this form of anthrax reflects the lack of facilities able to make the diagnosis in these areas. The spectrum of disease, ranging from subclinical infection to death, has not been fully recognized. In some community-based studies, cases of gastrointestinal anthrax outnumbered those of cutaneous anthrax. The oropharyngeal variant, in particular, is unfamiliar to most physicians. The clinical features of oropharyngeal anthrax include fever and toxemia, inflammatory lesion(s) in the oral cavity or oropharynx, enlargement of cervical lymph nodes associated with edema of the soft tissue of the cervical area, and a high case-fatality rate. Awareness of gastrointestinal anthrax in a differential diagnosis remains important in anthrax-endemic areas but now also in settings of possible bioterrorism.

The epidemiology of human anthrax has been described as agricultural and industrial (1). In the agricultural setting, infections occur from exposure to *Bacillus anthracis* spores on the skin or the mucosal surfaces of the gastrointestinal (GI) tract. Primary infections of the respiratory tract are rare in agricultural settings. Generally, reports state that the cutaneous form of anthrax is much more common than the GI form (2,3). We propose that the apparently overwhelming predominance of the cutaneous form of anthrax is rather a reflection of the difficulty of diagnosis of the GI form.

 GI anthrax may be diagnosed on the basis of epidemiology or microbiologic, pathologic, or serologic testing. Serologic diagnosis is available only in a few research laboratories; pathologic evaluation requires surgery or necropsy in an appropriate hospital; microbiologic testing requires at least microscopy and preferably bacterial culture capacity; and epidemiology requires a level of suspicion and an ability to properly perform outbreak investigations. Herbivores, which provide most of the human exposure risk for anthrax, become infected in rural parts of the world where spores in the soil perpetuate endemicity. Mild cases of gastroenteritis attract little attention, and people with severe infections, leading to death within 2–3 days, may never reach a medical facility.

Areas endemic for anthrax exist in all continents containing tropical and subtemperate regions. In the English published reports, deaths from GI anthrax have been reported in Thailand (4–6), India (7,8), Iran (9–11), Gambia (12), and Uganda (13). Anthrax-contaminated beef from a locally infected cow was eaten in Minnesota in 2000. Cooking the meat may have prevented human cases (14). Despite this wide distribution of endemicity, no large series of pathologically described cases exists. Based on limited reports of GI anthrax, the disease spectrum ranges from the asymptomatic to the fatal, by shock or sepsis. When swallowed, anthrax spores may cause lesions from the oral cavity to the cecum. Ulcerative lesions, usually multiple and superficial, may occur in the stomach, sometimes in association with similar lesions of the esophagus and jejunum (4,5,15). These ulcerative lesions may bleed; hemorrhaging in severe cases may be massive and fatal (4,5).

Reported cases indicate that lesions farther down the GI tract, in the mid-jejunum, terminal ilium, or cecum, tend to develop around a single site or a few sites of ulceration and edema, more analogous to cutaneous lesions. These lesions may lead to hemorrhage (6,16), obstruction (10,17,18), perforation (17,18), or any combination of these. Ascites may complicate GI anthrax (6,7,9,11,18,19). In some patients, the fluid shift from the vascular compartment leads to shock and death (6,7,9).

The pathologic examination of anthrax lesions with entry via the GI tract shows that the mucosa is always involved, as are regional lymph nodes, which are enlarged and hemorrhagic (15). The GI tract may also be involved after disseminated infection in pulmonary and sometimes cutaneous cases (20–22). In this situation, the localization is in the submucosa as a result of its blood flow, and the mucosa and regional lymph nodes become involved only secondarily.

These reports are biased toward the hospitalized patients with severe cases. Thorough epidemiologic reports are scarce. One informative description of an outbreak investigation and response comes from Uganda (13). Gastroenteritis developed and death occurred in 155 persons who feasted on an infected zebu (Asian ox); 2 days after exposure, the incident was reported to authorities who flew in a multiministry team the next day. Gastroenteritis developed in most (92%) of those exposed within 15–72 hours. All nine deaths were in children and occurred in the first 2 days; all 12 asymptomatic cases were in adults. Authorities referred 134 symptomatic people to the hospital for rehydration and treatment with antibiotics; all recovered. Thus, for most people exposed, the syndrome was gastroenteritis; a differential diagnosis would not normally have included anthrax. The age differences suggest that in this setting previous exposure may have occurred, leaving some adults with partial immunity.

Another community anthrax outbreak of note was investigated by officers from the Field Epidemiology Training Program of the Thai Ministry of Public Health (23). From January through June 1982, an outbreak of human anthrax was recognized in two districts in Udon Thani Province in northeastern Thailand after an outbreak in cattle that killed 36 water buffalos (*Bubalus bubalus*) and 7 cows and bulls. Of the 102 patients, 28 had cutaneous anthrax and 74 gastrointestinal anthrax. The only symptom in 67 of these 74 patients with gastrointestinal anthrax was acute diarrhea (i.e., gastroenteritis). The other seven patients had additional symptoms of nausea, vomiting, abdominal distention, and severe abdominal pain. Three patients died; the case-fatality rate was 4%. Thus, in these two community-based studies, the number of patients with GI anthrax far outnumbered those with the cutaneous form of the disease.

In addition to lack of epidemiologic, microbiologic, pathologic, and serologic expertise and facilities in the rural settings where anthrax outbreaks take place, the oropharyngeal form is underreported because few physicians are aware of the disease. Only six publications in the MEDLINE database report anthrax lesions in the mouth or oropharynx (24–29). Davies described a major epidemic of anthrax involving >9,000 patients in Zimbabwe from 1978 to 1980 (24). He cited four cases in which lesions were on the tonsil and the tongue but provided no details. We reported an outbreak of human anthrax after anthrax was found in water buffaloes in March–April 1982 in Chiang Mai, northern Thailand (25). A total of 52 cases of cutaneous anthrax and 24 cases of oropharyngeal anthrax were recognized in humans. All patients with oropharyngeal anthrax had recently eaten water buffalo meat. The mean incubation period was 42 hours (range 2–144 hours). All but one patient were admitted to the hospital. All patients sought medical attention because of painful neck swelling, and all but one complained of fever. The other common symptoms were sore throat, dysphagia, and hoarseness. The neck swelling was usually marked and was caused by enlargement of cervical lymph nodes and soft tissue edema (Figure 1). Mouth lesions were located on the tonsils, posterior pharyngeal wall, or the hard palate. In severe cases, the tonsillar lesions extended to involve the anterior and posterior pillars of fauces, as well as the soft palate and uvula. Early lesions were edematous and congested. By the end of the first week, central necrosis and ulceration had produced a whitish patch (Figure 2). In the second week, this patch developed into a pseudomembrane covering the ulcer (Figure 3). Diagnosis could be made by culture taken from the lesion in the mouth. A Gram stained smear from the lesion showed numerous polymorphonuclear leukocytes and gram-positive bacilli. Studies of serum antibody to anthrax antigens confirmed the diagnosis (27). Despite hospital admission and antibiotic treatment, 3 of the 24 patients died, for a case-fatality rate of 12.4%. In 1986, Doganay and colleagues reported six patients from Turkey with essentially the same clinical syndrome (28). The case-fatality rate in that study was 50%. The most recent case report, also from Turkey, was in 1993 (29).

**Figure 1:**
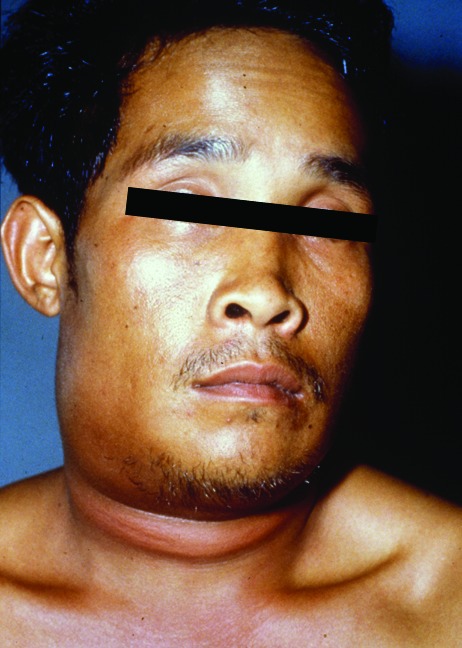
A 29-year-old man, 1 day after the onset of symptoms of oropharyngeal anthrax. Marked and painful swelling of the right side of the neck was present.

**Figure 2:**
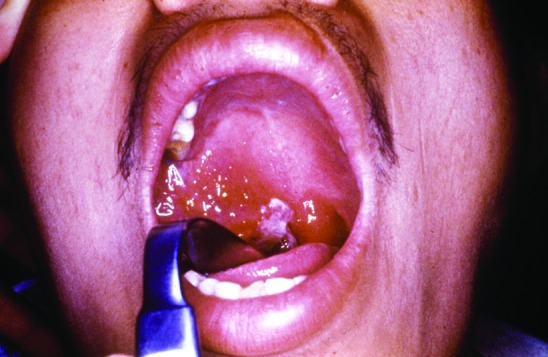
A 27-year-old man, 5 days after the onset of symptoms of oropharyngeal anthrax. Edema and congestion of the right tonsil extending to the anterior and posterior pillars of fauces as well as the soft palate and uvula were present. A white patch had begun to appear at the center of the lesion.

**Figure 3:**
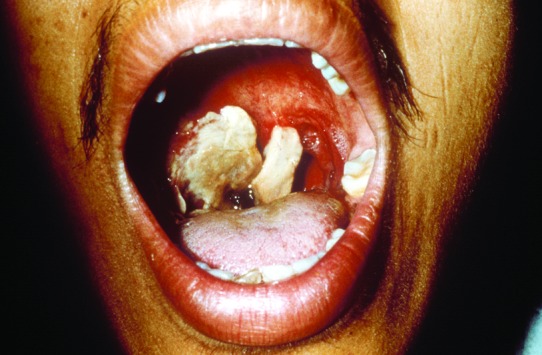
The same patient as in Figure 2. This picture is 9 days after the onset of symptoms of oropharyngeal anthrax. The white patch had developed into a pseudomembrane covering the lesion.

When cattle die of anthrax, the bacteremia is massive, and manifestations of the infection are visible to the butcher. In some settings, people may eat meat they know to be contaminated, as was the situation in a poor village of Harijans (“untouchables”) in India (8). More common may be the situation in which meat is sold to those unaware of the animal disease as was the case in the Chiang Mai outbreak (25). Only those who eat dishes that are raw or undercooked are exposed to infectious material. Disease is likely related to a dose of viable spores and the immune state of host. In contrast to the extreme susceptibility of cattle to this infection and its bacteremia, studies in chimpanzees suggest that primates are relatively resistant (30).

While the biowarfare and bioterrorist development of anthrax has focused on inhalation, ingestion has been considered as well. The Japanese experiments in China during the 1930s and 1940s included attempts to poison children with chocolate impregnated with anthrax (31). More recently, the apartheid government of South Africa had developed biological weapons, including another attempt at anthrax-containing chocolate (32). Given the large community outbreak of salmonellosis caused by an intentional contamination of restaurant salad bars the in United States by the Rajneeshees (33), awareness of the potential for GI anthrax due to bioterrorism is important.

In conclusion, GI anthrax is probably greatly underreported in rural disease-endemic areas of the world. The spectrum of disease, ranging from no symptoms to death, has not been fully appreciated. Awareness of anthrax in a differential diagnosis remains important in disease endemic-areas and also in settings of possible bioterrorism.
